# Ultrasound-Guided Reduction of Distal Radius Fractures 

**Published:** 2016

**Authors:** Anita Sabzghabaei, Majid Shojaee, Ali Arhami Dolatabadi, Mohammad Manouchehrifar, Mahdi Asadi

**Affiliations:** 1 Emergency Department, Loghman Hakim Hospital, Shahid Beheshti University of Medical Sciences, Tehran, Iran.; 2 Emergency Department, Imam Hossein Hospital, Shahid Beheshti University of Medical Sciences, Tehran, Iran

**Keywords:** Ultrasound, reduction, distal radius fracture, emergency department

## Abstract

**Introduction:**

Distal radius fractures are a common traumatic injury, particularly in the elderly population. In the present study we examined the effectiveness of ultrasound guidance in the reduction of distal radius fractures in adult patients presenting to emergency department (ED).

**Methods:**

In this prospective case control study, eligible patients were adults older than 18 years who presented to the ED with distal radius fractures. 130 consecutive patient consisted of two group of Sixty-Five patients were prospectively enrolled for around 1 years. The first group underwent ultrasound-guided reduction and the second (control group) underwent blind reduction. All procedures were performed by two trained emergency residents under supervision of senior emergency physicians.

**Results:**

Baseline characteristics between two groups were similar. The rate of repeat reduction was reduced in the ultrasound group (9.2% vs 24.6%; P = .019). The post reduction radiographic indices were similar between the two groups, although the ultrasound group had improved volar tilt (mean, 7.6° vs 3.7°; P = .000). The operative rate was reduced in the ultrasound groups (10.8% vs 27.7%; P = .014).

**Conclusion:**

Ultrasound guidance is effective and recommended for routine use in the reduction of distal radius fractures.

## Introduction:

Colles’ fracture is the most common wrist fracture in adults patients (1). It is a transverse fracture of the distal radial metaphysis, which is dorsally displaced and angulated, causing the classic “dinner fork” deformity seen on physical examination. Displaced distal radius fractures are usually managed with closed reduction under local or regional anesthesia, or procedural sedation and analgesia (PSA). Post-reduction radiographs are then obtained to assess the adequacy of the reduction. However, multiple inadequate reductions under blind manipulation can result in prolonged anesthesia time, increased sedation complication, increased radiation exposure, patient discomfort and additional investment of personnel, time and resource. Using ultrasonography (US) to guide reduction could improve these shortcomings (2)**.** Ang and co-workers supported the effective role of US guidance and recommended it as a routine technique in the reduction of distal radius fractures (3). Also previous studies showed that US has a good sensitivity and specificity in evaluation of long bone and wrist fracture (4-7). Narihito K. et al also suggest that US assistance can aid reduction of distal radius fractures as well as fluoroscopy (8). Based on diagnostic procedure, US also considered as a safe and reliable tool compared to X-ray diagnosis in juvenile fractures (9). Base on the above-mentioned, the purpose of our study was to assess the accuracy of ultrasound in monitoring closed reduction of distal radius fractures.

## Methods:

***Study design and setting***

This case control study was carried out during May 2012 to December 2013 in the emergency department (ED) of Haft-tir educational Hospital, Tehran, Iran. The study protocol was approved by ethical committee of Shahid Beheshti University of Medical Sciences. We sought to compare the results of US guided Colles’ fracture reductions, with the traditional technique of blind manipulation. The written informed consent was full fielded by all patients.

***Participants***

The patients with diagnosis of distal radius fracture (colles) undergoing US guided reduction were enrolled around a one-year period, under a convenience sample. All patients with age <18 years, open fracture, intra-articular step off > 2 mm, neurovascular compromise, volar tilt > 0 ˚, and poor compliance were excluded. 

***Procedure***

In this study, fracture reduction was done under procedural sedation-analgesia by fentanyl 1 µgr/kg and propofol 0.1 mg/kg. 130 eligible patients were categorized in each group for one years. At presentation to ED, anteroposterior (AP) and lateral (LAT) wrist x-rays were done to fracture confirmation. The attending emergency physician performed all reduction procedures and ulrasonographies. Two trained junior emergency residents performed traction-counterattraction at the three first finger and the arm under supervision of senior emergency physician. 7.5 MHz linear array US probe (SonoScape SSI-5500BW) was used to examine the fracture site by orienting the probe along the longitudinal plane on the dorsal and radial aspects of the radius. During the reduction, US views may be repeated as necessary until aligning the proximal and distal cortices into as straight a line as possible, seen in both AP and LAT views. The other group underwent reduction by the same condition but without US guidance. After reduction and immobilization, AP and LAT control x-rays were obtained for two groups. The accepted criteria for successful reduction were: volar tilt > 0 ˚, radial inclination angle of 15-25 ˚, and radial height > 5 mm. Another attempt was made in the cases of unsuccessful reduction. Final decisions for operative or conservative management were made by the orthopedic surgeon at either inpatient or outpatient settings. 65 patient underwent US guided and 65 cases underwent blind reduction. Two residents of was trained about 20 hours regarding performance of US and fracture reduction. 

***Statistical analysis***

SPSS 20 (SPSS, Chicago, Il, USA) was used to analyze the data. Pre- and post-reduction x-ray criteria such as volar tilt, radial inclination angle, radial height; number of attempts for reduction; successful reduction rate; and need for open reduction in operating room were compared between 2 groups.The Student t test was used to compare the difference in means between the groups and the χ 2 and Fisher exact tests were used to compare the different rates between groups. Results were considered statistically significant at the P < 0.05 level.

**Table 1:** **:** Baseline Characteristics of two studied groups

	**Ultrasonography (%)**	**Blind Manipulation (%)**	**P**
**Age, mean (SD):**	36.5 (15.8)	38.6 (17.1)	0.46
**Sex**			
Male	53 (81.5)	51 (78.5)	0.51
Female	12 (18.5)	14 (21.5)	
** Mechanism of trauma**			
Accident	22 (33.8)	25 (38.5)	0.80
		
Falling	40 (61.5)	38 (58.5)	
Direct Trauma	3 (4.6)	2 (3.1)	
**Side of fracture**			
Right	32 (49.2)	33 (50.8)	0.81
Left	32 (49.2)	30 (46.2)	
Right and Left	1 (1.5)	2 (3.1)	
**Fracture indices**			
Volar Tilt	-21.4 (13.1)	-19.1 (14.3)	0 .325
Radial Inclination 1	6.12 (6.3)	7.0 (5.3)	0 .346
Radial Height 1	8.0 (2.0)	7.8 (2.2)	0 .512

**Table 2:** Post reduction X-ray fracture indices between two groups

**Fracture Indices**	**Ultrasonography (%)**	**Blind Manipulation (%)**	**P**
**Volar tilt **	7.6 (5.2)	3.7 (6.0)	< 0.001
**Radial inclination **	18.8 (4.00)	18.4 (4.08)	0 .559
**Radial height **	10.1 (2.4)	9.4 (2.7)	0 .181

**Table 3:** Comparison of reduction outcome between two groups

	**Ultrasonography (%)**	**Blind Manipulation (%)**	**P**
**Reduction quality **			
Accepted	60 (92.3)	51 (78.5)	0.025
Non-accepted	5 (7.7)	14 (21.5)	
**Number of reduction **			
1	59 (90.8)	49 (75.4)	0 .019
≥ 2	6 (9.2)	16 (24.6)	
**Type of fixation **			
Casting	58 (89.2)	47 (72.3)	0.014
ORIF	7 (10.8)	18 (27.7)	

ORIF: open reduction and internal fixation.

## Results:

 130 patients with colles’ fracture were divided to two equal groups of US guided and blind fracture manipulation. Table 1 demonstrates the baseline characteristics’ of studied participants. The most common cause of injury was falling on outstretched hand (60%). As table 1 show there was no significant difference regarding sex, age, trauma mechanism, side of injury, and initial fracture indices between groups. Table 2 shows the result of pre and post reduction fracture indices. The post reduction radiographic indices were similar between the two groups, except for volar tilt (mean, 7.6° vs 3.7°; P < 0.001). The delta volar tilt in the US group was 29◦ compared to 22.8◦ for the other group. Table 3 compares the measured outcomes between two groups. The need for further attempt was significantly reduced in the ultrasound group (6 (9.2%) vs. 16 (24.6%); P = .019). The need for open reduction was significantly reduced in the US groups (7 (10.8%) vs. 18 (27.7%); P = .014).

## Discussion:

In this study, repeated attempts at reduction were significantly reduced by using US guidance. There was significantly improvement in volar tilt and decrease of operative rate. Most patients with a displaced distal radius fracture are initially managed with closed reduction under ﬂuoroscopy or without imaging assistance in the ED (8). Fluoroscopy is not readily available in all EDs and the patient and physician are exposed to radiation. Reduction without imaging guidance result in multiple attempt, more need for post reduction radiograph, increased patient discomfort, and radiation exposure. Restoring the volar angle itself result in better functional outcome and is an important indicator for surgery. Obviously, ultrasound cannot directly measure radial height, radial inclination, or volar tilt, but alignment of the distal and proximal bony fragments of the radius in two planes can indirectly predict amount of these indices. US has a good sensitivity and specificity in evaluation of long bone (4, 5) and wrist fracture (6, 7). US also is a useful tool in evaluation and reduction attempt in infants (10-13). Multiple studies have been declared the successful utility of US in reduction of different type of fractures. Ang et al. stated that US guidance is effective and recommended it for routine use in the reduction of distal radius fracture (8). Shiang-Hu et al. reported that US group had improved volar tilt (mean, 5.93° vs 2.61°; P = .048). They also reported that operative rate was also reduced in this group (4.9% vs 16.7%; P = .02) (3). In the current study the accepted (successful) reduction was better in ultrasound group 92% (60/65) than blind group 78% (51/65). Narihito K. et al reported successful reduction of 95% (41/43) in ultrasound group versus 68%, that was in line with our findings (15/22) (8). Sono-guided reduction is an accurate, simple, and safe technique that provides the considerable advantage of real-time observation. The advantages of US guidance over blind manipulation are decrease the number of reduction attempts and consequently fewer traumas to the surrounding soft tissues (3). The greatest value in US-guided reduction may lie in its ability to provide the practitioner with immediate imaging of bony alignment after each reduction maneuver, therefore decreasing the need for repeat procedural sedations and removal and reapplication of the splint (3, 7, 14). Finally, while US has some limitations that prevent it from completely replacing conventional radiography, it can facilitate the reduction and prevent repeated reduction attempts. There were some limitations of our study. Due to our design limitations, we did not study whether the use of US could decrease the time spent in the ED or not. Also as our limitation of resource and overcrowding, we couldn’t use finger trap for traction and instead of it two person performed traction-counterattraction at the three first finger and the arm. However, it was performed similarly in both ultrasound and control groups.

## Conclusion:

It seems that, ultrasonography is a suitable guidance tools for wrist fracture reduction and is recommended for routine use in real-time monitoring of close reductions.

## Conflict of interest:

None

## Funding support:

None

## Authors’ contributions:

All authors passed four criteria for authorship contribution based on recommendations of the International Committee of Medical Journal Editors.
